# High Hydrostatic Pressure Assisted by Celluclast^®^ Releases Oligosaccharides from Apple By-Product

**DOI:** 10.3390/foods9081058

**Published:** 2020-08-05

**Authors:** Rocío De la Peña-Armada, María José Villanueva-Suárez, Pilar Rupérez, Inmaculada Mateos-Aparicio

**Affiliations:** 1 Departamentode Nutrición y Ciencia de los Alimentos, Facultad de Farmacia, Universidad Complutense de Madrid, Plaza Ramón y Cajal s/n, 28040 Madrid, Spain; rociojim@ucm.es (R.D.l.P.-A.); mjvilla@ucm.es (M.J.V.-S.); inmateos@ucm.es (I.M.-A.); 2 Departamento de Metabolismo y Nutrición, Instituto de Ciencia y Tecnología de Alimentos y Nutrición (ICTAN), Consejo Superior de Investigaciones Científicas (CSIC), José Antonio Novais 10, Ciudad Universitaria, 28040 Madrid, Spain

**Keywords:** apple by-product, valorisation, green technology, HHP, cellulase, dietary fibre, polysaccharides, oligosaccharides, prebiotics

## Abstract

A novel and green procedure consisting of high hydrostatic pressure (HHP) aided by a commercial cellulase (Celluclast^®^) has been applied to valorise the apple by-product, a valuable source of dietary fibre but mainly composed by insoluble fibre. Optimal conditions for solubilisation of dietary fibre were first determined at atmospheric pressure as 2% (*w*/*v*) of substrate concentration and 20 Endo-Glucanase Units of cellulase. Monitoring of polysaccharides and oligosaccharides released from apple by-product was carried out by means of a newly validated HPLC method with refractive index detector. A synergistic effect was observed when the combined HHP plus cellulase treatment was used. Thus, the application of 200 MPa at 50 °C for 15 min enabled a significant increase in the release of water-soluble polysaccharides (1.8-fold) and oligosaccharides (3.8-fold), as well as a considerable decrease in the time required (up to 120-fold), compared to control at 0.1 MPa. Therefore, this technology could be a promising alternative approach to transform an industrial by-product into a novel rich-in-oligosaccharide food ingredient and a step forward into shaping the world of prebiotics.

## 1. Introduction

Apple (*Malus* spp.) represents one of the most widespread fruit crops in the world. In 2018 its global production exceeded 85 million metric tonnes according to FAO statistics. Besides, a significant part of apple harvest, between 25 to 30% [1], is intended to process and transform, mainly, into apple juice or cider production, in tandem, a solid residual material known as apple by-product [2]. Apple by-product consists of the apple peel, seeds, stalks and some pulp being equivalent to 25% of the fresh fruit weight. Hence, the expected amount of underused by-products is up to 3.5 million tons per year approximately [3], which leads to an expensive and complex procedure for an effective and safe final disposal [4]. Hawken and collaborators in 2018 reported that the third most effective solution for climate change mitigation is reducing food waste [5]. Accordingly, by-product valorisation might produce economic and health benefits for the society, while using the existing natural resources to develop new value-added products [6]. In this regard, apple by-product has been determined as an interesting source of bioactive compounds due to its nutritional composition including dietary fibre [4,6]. It is widely acknowledged that soluble dietary fibre fraction exhibits a potential prebiotic effect by means of its content in specific substances, such as oligosaccharides, with interesting fermentability profile and their certain interaction with colonic microbiota [7,8]. Oppositely, apple pomace, presents a higher water-insoluble carbohydrates amount, namely, hemicellulose and cellulose, than soluble-water carbohydrates quantity [6,8]. However, preclinical studies have demonstrated a positive effect on lipid metabolism and gastrointestinal function of apple by-product, suggesting its potential use as a functional food and/or food additive [6,9,10,11]. Therefore, it would be of industrial interest to develop a resource-efficient process to reintroduce this derived product into the human food chain since, currently, scientists are attempting to synthesize prebiotics on an industrial scale [12]. The application of suitable technologies to transform apple by-product, into a safe and good quality output could be of relevance. The perishable nature of the material (65.9% moisture content) is a handicap regarding the stability factor. Thus, to choose a system in which extending shelf-life, preserving or even improving the nutritional and functional characteristics of apple by-product, could be of primary importance.

Presently, numerous strategies have been explored towards promoting sustainable techniques, alternative to conventional methodologies due to its proven higher efficiency and environmental respectfulness [13]. Enzyme-based methods have been reported as an alternative green novel approach for by-product valorisation [14] comprising its application for enhancing pectin extraction from various derived products, among which apple by-product is included [14,15,16,17,18]. Likewise, digestion with Celluclast^®^, a food-grade enzyme (cellulase or endo-β-glucanase) with high cellulo- xylano-, and mannolytic activities, has demonstrated a comparable or higher pectin extraction capacity than the acid treatments commonly used [19]. Nevertheless, few studies have focused on the potential utilisation of this methodology to produce compounds that could work as selective growth-promoting substrates [20,21] and therefore, further elementary research on enzymatic hydrolysis of agro-industrial by-products is required [14]. The integration of enzyme-based method with different emerging technologies such as, high hydrostatic pressure (HHP), has been proposed for making up enzyme technology shortfalls such as high solvent consumption or long incubation times, and also, for intensifying the extraction of bioactive compounds from plant based materials, as previously reported for the increase in pulp polysaccharides yield from longan fruit [22] or solubilisation of dietary fibre in okara soybean by-product [23,24,25,26]. HHP is a non-thermal processing technology in which, high pressure of 100–600 MPa is applied by using a liquid, typically water, as the pressure transfer medium to the surface and interior of the food matrix, leading to some structural changes that may increase cell permeability [27].

However, to the best of our knowledge, the combined HHP and food-grade enzymes treatment has not been explored previously in apple by-product. Therefore, the aim of this study was to appraise the effect of high pressure assisted by Celluclast^®^ on soluble polysaccharides and oligosaccharides from apple by-product and to determine optimal conditions for soluble dietary fibre maximization. For that purpose, a three-variable battery of enzymatic experiments was designed, release of soluble carbohydrates amount was monitored by an HPLC-RID novel method and outcomes were analysed in order to decide the finest optimal substrate concentration and enzyme dose combination for HHP enzyme assisted assays.

## 2. Materials and Methods

### 2.1. Raw Material and Celluclast^®^ 1.5 L Enzyme

Apple by-product obtained from Golden Delicious variety was provided by Zucasa (Zumos Catalano Aragoneses S.A., Huesca, Spain). Once in the laboratory, it was freeze-dried (LyoQuest Freeze Dryer, Telstar S.A. Madrid, Spain), ground into fine powder (particle size <1 mm) and stored in a dry atmosphere. Previously to all analyses, apple by-product was hydrated in distilled water at room temperature with constant shaking Heidolph Reax 2 (speed from 1–9) overnight to simulate raw fresh material conditions.

Enzymatic treatment was performed with Celluclast^®^, a commercial food-grade cellulase (endo-β-glucanase) from *Trichoderma reesei* (Novozymes, Bagsvaerd, Denmark).

### 2.2. Optimization of Celluclast^®^ Treatment

Enzyme procedure was optimized at atmospheric pressure prior to being combined with HHP technology. For that purpose, enzymatic activity of Celluclast^®^ was assessed at different substrate and enzyme concentrations. Experimental design was based on previous studies of Celluclast^®^ application in apple by-product pectin extraction [19]. Three different variables were determinant for the release of soluble carbohydrates (oligo- and polysaccharides) from the insoluble dietary fibre of apple by-product during the enzymatic treatment with Celluclast^®^, namely, time 0.5–30 h, apple by-product concentration 0.5–4% (*w*/*v*) and cellulase enzyme dose 10–20 Endo-Glucanase Units (EGU). One EGU indicates the amount of Celluclast^®^ required releasing one μmol of glucose per minute, under the assay conditions. Apple by-product was hydrated (0.5%, 2% and 4% *w*/*v*) and processed with Celluclast^®^ 10 or 20 EGU. The incubation was carried out in a water bath at 50 °C with constant shaking. Aliquots were taken at 0, 0.5, 1, 3, 6, 8, 24 and 30 h. Time zero was assigned as the control. After incubation, samples were immediately shocked by heat to stop enzyme activity. Then, samples were cooled and filtered through 0.45 μm syringe filters (cellulose acetate, 25 mm diameter, Análisis Vínicos, Tomelloso, Toledo, Spain).

For this kinetic monitorization, uronic acid and reducing sugars were spectrophotometrically measured according to the colorimetric method of 3,5-dimethylphenol [28] and 3,5-dinitrosalicylic acid (DNS) procedure [29], respectively. Both methods were conveniently adapted for a microplate reader (Synergy™ HTX Multi-Mode, BioTek, Winooski, VT, USA). Briefly, for uronic acid quantification, NaCl/H_3_BO_3_ solution (60 μL) and concentrated H_2_SO_4_ (800 μL) were added to galacturonic acid standards (50–200 ppm) and samples (60 μL). After 10 min reaction time at 70 °C, samples were cooled down and consecutively mixed with 50 μL of 3,5-dimethylphenol colorimetric reagent. The spectrophotometric measurement was carried out at 400 and 450 nm after 15 min since the last reaction mixture. Regarding reducing sugars, DNS determination consisted of adding 50 µL of 3.9 M NaOH and 200 µL of 3,5-dinitrosalicylic acid colorimetric reagent to 100 µL of glucose standards (250–1000 ppm) and samples followed by agitation and 10 min reaction in a thermostatic bath at 100 °C. Finally, the resulting solution was diluted 1:4 (*v*/*v*) in distilled water and absorbance was measured at 550 nm.

Furthermore, polysaccharides (100–5.94 kDa MW) oligosaccharides (0.83–0.50 kDa MW) and simple sugars (0.34–0.18 kDa MW), derived from the enzymatic hydrolysis of apple by-product were quantified by HPLC-RID.

Multiple linear regression as a data analysis tool was used based on data obtained for polysaccharides, oligosaccharides, and simple sugars amount (*Y*) in g/100 g of dry matter. The polynomial equation included enzyme dose (*X*_1_) in EGU, time (*X*_2_) in hours and substrate concentration (*X*_3_) expressed as percentage (*w*/*v*) factors.

### 2.3. HHP Treatment Assisted by Celluclast^®^ Procedure

Pre-hydrated apple by-product (10% *w*/*v*) was treated with 92 EGU of Celluclast^®^ (1:40 enzyme:substrate, *v*/*w*) and HHP (pressures of 200, 400 and 600 MPa), at 50 °C, for 15 or 30 min. Conditions for substrate and enzyme concentrations were determined based on previous data analysis of the cellulase activity. Control was the sample at atmospheric pressure 0.1 MPa and 22 ± 1 °C. For that purpose, apple by-product samples plus the enzyme was placed in vacuum-sealed plastic bags (200 × 300 mm CRYOVAC, Ref. BB3255; Alfredo Martínez, Madrid, Spain) and consecutively, HHP was applied in a laboratory-scale high-pressure vessel (Stansted SFP 7100:9/2C equipment) sited in ICTAN (CSIC) in which the transmitting medium was water. Additionally, one more procedure was undertaken in absence of Celluclast^®^ enzyme in order to ascertain the unilaterally effect of HHP. The different pressure treatments were performed in duplicate. After the HHP assisted and non-assisted by Celluclast^®^ treatment an aliquot was taken, filtered through 0.22 µm and analysed by HPLC. HPLC-RID was used for high molecular weight carbohydrates and oligosaccharides determination, including identification by RT coincidence and quantification according to peak area, as well as, molecular weight estimation by using the logarithmic calibration curves. Furthermore, uronic acid and reducing sugars were assessed for data analysis and comparison with the enzymatic treatment.

### 2.4. High Performance Liquid Chromatography Instrument, Calibration and Validation

HPLC system was equipped with a Refractive Index Detector (RID) and the analysis of carbohydrates was performed on a Rezex™ RSO-Oligosaccharide Ag 4%, LC Column 200 × 10 mm, preceded by a Rezex™ RSO-Oligosaccharide Ag 4%, LC Guard Column 60 x 10 mm (Phenomenex^®^, Torrance, California, CA, USA). Milli-Q^®^ water was used as mobile phase, at a flow rate of 0.3 mL/min. The column was maintained in a thermostatic oven at a constant temperature of 65 °C. Injections of standards and samples (5 μL) were made after filtering through 0.22 μm filters for aqueous solutions (Nylon syringe filters, 0.22 µm diameter Branchia, Labbox, Barcelona, Spain). Ultrapure water (Resistivity 18.2 MΩ cm at 25 °C; Milli-Q Integral 5 Water Purification System from Millipore, Merck KGaA, Darmstadt, Germany) was used for preparing dilutions, as well as, mobile phases for chromatography analysis.

Thirteen carbohydrate standards with different average molecular weight (MW) were used for retention time (RT) and calibration curve calculation: Pullulan 100 (100 kDa), Pullulan 50 (50 kDa), Pullullan 20 (20 kDa) and Pullulan 10 (10 kDa) from a Shodex pullulan standard P-82 kit were obtained from Waters, Madrid, Spain. Inulin (5.94 kDa), Verbascose (0.83 kDa), Stachyose (0.67 kDa), Cellotriose (0.50 kDa), Raffinose (0.50 kDa), Glucose (0.18 kDa) and Fructose (0.18 kDa) were obtained from Sigma, (Alcobendas, Madrid, Spain). Cellobiose (0.34 kDa) and Sucrose (0.34 kDa) were obtained from Merck (Darmstadt, Germany). Each standard was injected in triplicate at diverse concentrations (0.125–1 mg/mL) into the HPLC-RID. Linear regression standard curves were calculated using, the standard concentration *versus* the area obtained from the RID (in Nriu *s) and, the linearity (*R*^2^), were assessed. Furthermore, equations for high molecular weight carbohydrates (HMWC) and for oligosaccharides molecular weight estimations were calculated. These equations were used estimating the average molecular weight of standards for validation.

Precision of the HPLC-RID method was assessed by intra- and inter-day repeatability assay. Results were expressed as relative standard deviation (RSD) percent of the retention time. Moreover, the limits of detection (LOD) and quantitation (LOQ) were calculated according to the signal-to-noise ratio from standard solutions with known low concentrations (10 µg/mL) and blank solutions. For LOD calculation, signal-to-noise ratio equals 3. For LOQ, signal-to-noise ratio equals 10 [30].

### 2.5. Statistical Analyses

Statgraphics18 was used for detailed statistics. Results were expressed as mean values ± standard deviation. Comparison of means was performed by one-way analysis of variance (ANOVA) with a significance level of *p* < 0.05. Consecutively, Duncan multiple comparisons test was employed to determine whether mean values were significantly different between treatments (*p* < 0.05).

## 3. Results

### 3.1. Optimization of Enzymatic Treatment with Celluclast^®^

**Figure 1:**
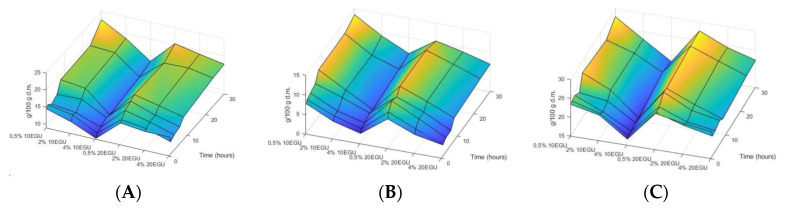
HPLC-RID analysis of (**A**) polysaccharides, (**B**) oligosaccharides (**C**) and simple sugars released from apple by-product during enzymatic treatment with Celluclast^®^ at atmospheric pressure. Data are expressed as g/100 g dry material. EGU: Endo-Glucanase Units.

Celluclast^®^ treatment consisted of using different apple by-product concentrations (0.5, 2 and 4%), at several enzyme doses (10 and 20 EGU), over a 30-h period of incubation. Monitorization was carried out by HPLC-RID analyses, whereby, it was possible to determine the aliquots carbohydrates composition. Celluclast^®^ 1.5L treatment produced a release of the soluble high molecular weight carbohydrates (HMWC) and oligosaccharides from the sample matrix. Nevertheless, differences were noted in the cellulase kinetics, specifically, both enzyme doses (10 and 20 EGU) allowed the release of HMWC and low molecular weight carbohydrates (LMWC) at 0.5% and 2% apple by-product concentration being such an increase significantly greater than the observed at 4% apple by-product concentration. Moreover, at short incubation dwell time, carbohydrates increase at 0.5% and 2% apple by-product concentration and 20-cellulase units were significantly higher (*p* < 0.05) than at 10-cellulase units treatment. In fact, within 3 h incubation, the amount of HMWC was 2.0-fold higher than the control, whereas oligosaccharides quantity was 16.6 to 22.7-fold higher compared to the non-treated sample (Figure 1). Indeed, the extraction of HMWC and oligosaccharides reached over 80% of total carbohydrates. The multiple regression analysis was used to fit three linear equations according to polysaccharides (Equation (1)), oligosaccharides (Equation (2)) and simple sugars (Equation (3)) data.

**Figure 2:**
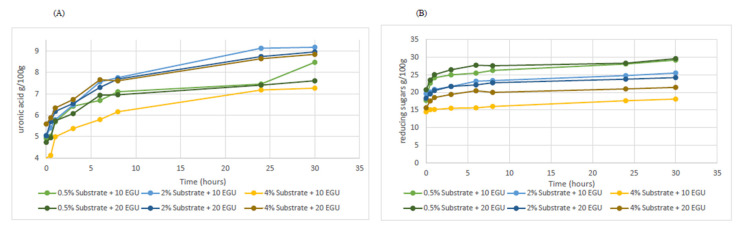
Determination of (**A**) uronic acids and (**B**) reducing sugars released from apple by-product during enzymatic treatment with Celluclast^®^ at atmospheric pressure. Data are expressed as g/100 g dry material.

Regarding uronic acid quantification, after 30 h incubation, the amount resulted almost double than that at time zero. A slight increase tendency in uronic acids was observed for the experiments developed at 2% compared to those at 0.5% and 4% substrate concentration assisted by, either, 10 or 20 U of cellulase. Moreover, it was noted a significant (*p* < 0.05) raise of reducing sugars for 0.5% substrate concentration treatment at both, 10- and 20-EGU enzyme dose compared to the other treatments (Figure 2).

[*Y* = 12.41 + 0.20 *X*_1_ + 0.39 *X*_2_ − 1.14 *X*_3_] (1)

[*Y* = 7.85 + 0.04 *X*_1_ + 0.39 *X*_2_ − 1.60 *X*_3_] (2)

[*Y* = 25.09 + 0.03 *X*_1_ + 0.17 *X*_2_ − 2.37 *X*_3_] (3)

*X*_1_ = Enzyme dose in EGU
*X*_2_ = Time in hours
*X*_3_ = Substrate concentration in percentage (*w*/*v*)

The adjusted equations included data collected until 8 h-time because of linearity. Equations were adequate and a good representation of the behaviour of the system, with *R*^2^ values for polysaccharides, oligosaccharides and simple sugars quantity, of 0.85, 0.93 and 0.92, respectively.

### 3.2. HHP Assisted by Celluclast^®^ Treatment of Apple By-Product

**Figure 3:**
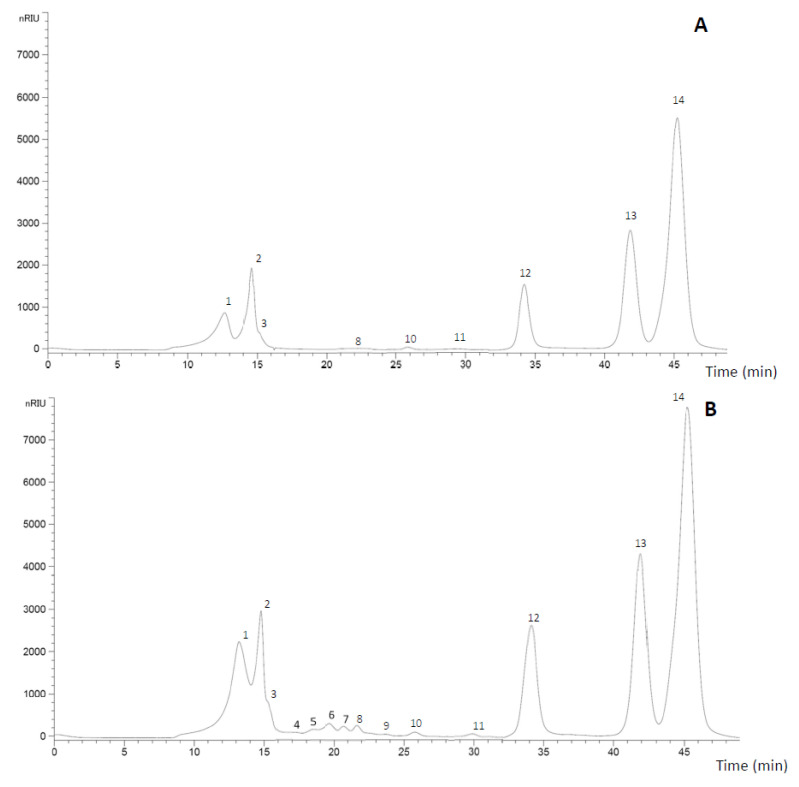
HPLC-RID chromatograms of apple by-product: (**A**) control and (**B**) samples treated with HHP (200 MPa) for 15 min assisted by Celluclast^®^ (92 EGU). Peaks 1–5 were estimated as polysaccharides or high molecular weight carbohydrates (HMWC). Peaks 6–14 were estimated as oligosaccharides and simple sugars or low molecular weight carbohydrates (LMWC).

**Figure 4:**
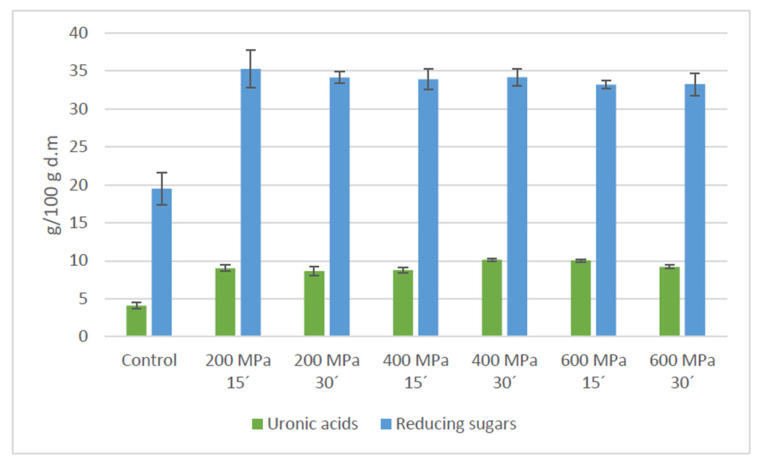
Determination of uronic acids and reducing sugars released from apple by-product by Celluclast^®^ (92 EGU) under high hydrostatic pressure at 200, 400 and 600 MPa for 15 and 30 min, respectively. Data are expressed as g/100 g dry material.

Data of soluble polysaccharide and oligosaccharide content of apple by-product simultaneously treated with HHP and Celluclast^®^ are presented in Table 1 and Figure 3. Peaks 1–5 were calculated as polysaccharides including all identified compounds with a molecular weight that ranged from 1005.94 kDa. Moreover, defined peaks from 6 to 14 were determined as components with a molecular weight between 0.83 and 0.18 kDa and estimated as oligosaccharides and simple sugars. Results showed a significant raise of the HMWC amount reaching values almost two times higher than the control. However, a similar outcome was noted when HHP was exclusively applied (Table 2). Besides, oligosaccharides were only increased in high pressure assisted plus cellulase treated samples by 2% to represent 13% of the total soluble carbohydrates. Regarding to disaccharide compounds, an increment of its quantity was observed after both, HHP and HHP assisted by Celluclast^®^ treatments, being in the latter case such growth higher. Furthermore, no significant composition differences were found among the high pressure plus cellulase treated samples despite of the diverse high-pressure conditions tested. An identical pattern was observed for the HHP non-assisted by Celluclast^®^ samples. Additionally, 3,5-dimethylphenol assessment determined a significant (*p* < 0.05) increase in uronic acids when treatment involved simultaneously high pressure and cellulase enzyme. Regarding reducing sugars quantity, determined by DNS method, results showed an increment (*p* < 0.05) that almost doubled values obtained for control (Figure 4).

### 3.3. HPLC Calibration and Validation Methodology

Different standards, including polysaccharide, oligosaccharide and monosaccharide with average MW ranging from 100 to 0.18 kDa, were used for the quantitative analysis of carbohydrates on the Rezex™ RSO-Oligosaccharide column. An equation for each standard was determined [Area in nRiu∗s = m (concentration in mg/mL) + b] and the linearity of the calibration curve was evaluated (Table 3). All the values obtained for *R*^2^ were above 0.998. Furthermore, regression standard curves for estimation of carbohydrates molecular weight were calculated, namely, for HMWC (Log (MW) = −2.1799 RT + 37.694) and for oligosaccharides and simple sugars (Log (MW) = −0.0315 RT + 3.623), whereby, the *R*^2^ values were 0.97 and 0.98, respectively. In addition, standards molecular weight was estimated, and results differed less than 10 kDa for polysaccharides and 0.06 kDa for oligosaccharides.

RID sensitivity was determined by LOD, varying from 1.22 to 3.52 µg/mL and LOQ range from 4.07 to 11.72 µg/mL. Detection and quantification limits resulted almost three times lower for HMWC than for oligosaccharides.

Besides, precision of the HPLC-RID method with RSO column was calculated based on the retention time replicability intra- and inter-day of the triplicate standards injected separately in consecutive days. Data from the first day were used for the intraday repeatability estimation. Results were obtained and percentage of relative standard deviation (RSD) was calculated (Table 3). Thus, data for RSD (%) did not exceed 0.03% for repeatability or 0.06% for inter-day precision.

## 4. Discussion

Recent advances on the sustainable management of food by-products have emphasised the use of food-grade enzymes as an interesting approach for conversion of agro-industrial wastes, into valuable bioproducts [31]. Enzymes are capable to degrade cell walls, as well as, different vegetable membranes, enabling the extraction of interesting health promoting substances such as pectins or oligosaccharides [8,18]. The different kinetics of cellulase treatments (Celluclast^®^) using two enzyme doses (10 and 20 EGU) on apple by-product at several concentrations (0.5, 2, 4%), and combined or not with HHP treatment, were monitored by the validated HPLC-RID method. Celluclast^®^ treatment accomplished at atmospheric pressure was able to produce soluble oligosaccharides from apple by-product and a significant increase (*p* < 0.05) in soluble polysaccharides concentration (Figure 1). However, numerous authors have reported the synergistic effect of high pressure and enzyme activity for bioactive compounds extraction [23,24,25,26,32,33], achieving, when applying both methodologies together, better results than by employing them separately. Hence, data analysis from Celluclast^®^ treatment performed at atmospheric pressure was to proceed in order to determine optimal substrate and enzyme conditions for further combined HHP and Celluclast^®^ treatment.

Results indicated that 10 and 20 EGU of enzyme were equally effective at 0.5 and 2% substrate concentration regarding HMWC release, whereas, during short incubations periods 20 EGU cellulase dose generated a greater amount of HMWC (Equation (1). In addition, 20 units of cellulase worked better than 10 units of the enzyme (Equation (2) regarding LMWC production (Figure 1) and uronic acid quantification (Figure 2) at the same substrate concentration. Furthermore, monitorization of the disaccharides content by HPLC-RID methodology (Figure 1) revealed an increment of its quantity (Equation (3), which corresponded, to an increase in cellobiose molecules removed from the hydrolysed cellulose network [34,35,36]. Data are in accordance with those obtained for reducing sugars determination assay (Figure 2), in which, the major increment results at 0.5% substrate concentration followed by 2% and 4%. Thus, even though, the oligosaccharides increment was higher at 0.5% substrate concentration, it was notable that increasing four times the apple by-product material (2% substrate concentration) and maintaining the enzyme dose (20 EGU) resulted in the same polysaccharides quantity and a similar oligosaccharides amount. Differences were accounted in less than one gram for oligosaccharides as compared with the highest reached values. Consequently, it seemed appropriate to appoint optimum conditions as 2% substrate concentration and 20 EGU enzyme dose due to the convenient substrate: enzyme proportion and the favourable carbohydrates solubilisation values obtained.

HHP assisted by Celluclast^®^ treatment was performed in order to maximize soluble polysaccharides and oligosaccharides due to the prebiotic effect associated to these components [6,7,11,32,33]. Results showed an increase in both, HMWC and LMWC, commensurate to previous analysis performed individually with high pressure or cellulase enzyme. By co-treatment, an increase in the water-soluble polysaccharides fraction equivalent to high-pressure individual process was noted (Table 2) and similar results were achieved when Celluclast^®^ at 0.5%, 2% or 4% substrate concentration and 20 EGU enzyme dose was applied for 30 h (Figure 1). Furthermore, uronic acid of the combined treatment, which corresponded to pectin solubilization, achieved analogous outcomes to cellulase treatment performed with 2% substrate concentration for 30 h (Figure 2 and Figure 4). Therefore, the application of high pressure, even at the minimum magnitude of pressure tested, namely, 200 MPa, for 15 min, diminished in more than 100 times the required period for reaching comparable results in HMWC by the Celluclast^®^ treatment at atmospheric pressure. Regarding oligosaccharides fraction, results for the simultaneous assay were akin to 2% apple by-product substrate concentration subjected to 20 units cellulase incubation for 6–8 h. Accordingly, HHP combined with an enzymatic treatment resulted more effective for releasing of soluble oligosaccharides than by conventional long-time cellulase incubation assays.

Moreover, a general interest within the scientific community exists regarding the need of specific techniques for quantifying indigestible carbohydrates of low molecular weight, otherwise known as, oligosaccharides [8]. According to our present results, a useful methodology has been developed for polysaccharides and oligosaccharides determination. As shown in Figure 3, the HPLC-RID chromatograms revealed clearly the increased of water-soluble carbohydrates amount for the combined treatment. The oligosaccharides quantity represented by peaks from 4 to 11, was enhanced when the Celluclast^®^ enzyme was added. According to the present results, cellulase can promote the breaking of the bonds of the cell wall molecules, therefore releasing cellulose-type oligosaccharides. Furthermore, the possible increase in the cell wall accessibility facilitates enzymatic activity on different carbohydrates that make up plant cell structures.

Optimal conditions for the HPLC method considering the column and detector used for the analysis, as well as, the expected outcomes for this technique, were set at 65 °C temperature and 0.3 mL/min flow after recording fine results for linearity, sensitivity, precision and accuracy parameters. In this regard, regression curves for each carbohydrate standard exhibited a good correlation coefficient (*R*^2^ ≥ 0.998) and a linear range from 0.25 mg/mL to 1 mg/mL. Furthermore, for MW estimation, due to the ample molecular weight range of the standards and the lack of linearity after the log MW *versus* log RT analysis, two different calibration curves were determined distinguishing on the one hand, the equation for MW estimation of polysaccharides and on the other hand for oligosaccharides. Besides, preciseness was evaluated for both, concluding with good results either for HMWC or for LMWC (Table 3). Moreover, RID showed to be sensitive enough for different MW carbohydrates determination, namely, LOD and LOQ values were approximately fifty-times lower than values described by Gómez-Ordóñez and col. [30] (2012) for polysaccharide standards (50–210 µg/mL and 160–310 µg/mL, respectively) and four-times lower than LOD and LOQ previously reported for ELSD detector (4.83–11.67 µg/mL) [15]. In addition, precision of the method was evaluated by repeatability of the retention time outcome for intra- and inter-day assay. Deliverables for RSD (%) below 0.06% currently indicated that the method had both good repeatability and inter-day precision.

## 5. Conclusions

A synergistic effect of high pressure aided by Celluclast^®^ enzyme treatment has been proved to hydrolyse the insoluble dietary fibre of apple by-product. This simultaneous treatment, in conjunction with a deployment of optimal conditions, namely, 2% (*w*/*v*) substrate concentration, 20 EGU of Celluclast^®^ enzyme dose and 200 MPa pressure for 15 min, was able to originate a 1.8-fold raise in water-soluble polysaccharides and a 3.8-fold increase in oligosaccharides content, as measured by the validated HPLC-RID method. Hence, a potential prebiotic product was obtained in a short period, establishing this dual process, as a promising approach for the valorisation of an insoluble dietary fibre-rich plant by-product. Further investigation into this enzyme treatment assisted by emerging technologies, such as HHP, on apple by-product, could promote the enhancement in polysaccharides and oligosaccharides and the development of high value-added products from a natural plant resource.

## Figures and Tables

**Table 1:** HPLC-RID analysis of water-soluble carbohydrates released from apple by-product after HHP treatment assisted by Celluclast^®^.

	RT (min)	Sample Time (min)	Control	200 MPa		400 MPa		600 MPa	
				15	30	15	30	15	30
Peak 1	13.20		3.63 ± 0.10 ^a^	8.51 ± 0.33 ^cd^	7.73 ± 0.38 ^b^	8.02 ± 0.22 ^bc^	8.97 ± 0.33 ^d^	8.62 ± 0.24 ^d^	8.49 ± 0.32 ^cd^
Peak 2	14.77		3.13 ± 0.05 ^a^	3.95 ± 0.51 ^e^	3.51 ± 0.10 ^b^	3.71 ± 0.10 ^c^	4.05 ± 0.05 ^d^	3.77 ± 0.14 ^c^	3.96 ± 0.14 ^d^
Peak 3	15.04		0.46 ± 0.07 ^a^	0.87 ± 0.08 ^d^	0.73 ± 0.07 ^c^	0.82 ± 0.02 ^cd^	0.89 ± 0.05 ^d^	0.58 ± 0.11 ^b^	0.56 ± 0.04 ^b^
Peak 4	17.21		Nd	1.01 ± 0.15 ^b^	0.84 ± 0.20 ^b^	0.74 ± 0.13 ^b^	0.77 ± 0.07 ^b^	2.16 ± 0.32 ^c^	2.13 ± 0.29 ^c^
Peak 5	18.57		Nd	1.32 ± 0.03 ^d^	1.08 ± 0.15 ^c^	1.01 ± 0.09 ^bc^	1.14 ± 0.08 ^cd^	1.15 ± 0.17 ^cd^	0.82 ± 0.14 ^b^
Peak 6	19.68		Nd	1.09 ± 0.15 ^e^	0.88 ± 0.07 ^de^	0.82 ± 0.06 ^cd^	0.94 ± 0.03 ^de^	0.70 ± 0.04 ^c^	0.52 ± 0.14 ^b^
Peak 7	20.74		Nd	0.73 ± 0.09 ^d^	0.59 ± 0.15 ^c^	0.50 ± 0.05 ^bc^	0.55 ± 0.03 ^c^	0.49 ± 0.02 ^bc^	0.41 ± 0.07 ^b^
Peak 8	21.69		0.27 ± 0.05 ^a^	0.72 ± 0.07 ^e^	0.68 ± 0.03 ^de^	0.59 ± 0.03 ^c^	0.64 ± 0.04 ^cd^	0.75 ± 0.06 ^e^	0.49 ± 0.18 ^b^
Peak 9	22.33		Nd	0.77 ± 0.10 ^d^	0.60 ± 0.07 ^bc^	0.56 ± 0.07 ^bc^	0.65 ± 0.07 ^cd^	0.52 ± 0.08 ^b^	0.49 ± 0.02 ^b^
Peak 10	25.81		0.27 ± 0.06 ^a^	0.89 ± 0.30 ^b^	0.68 ± 0.15 ^b^	0.60 ± 0.10 ^b^	0.63 ± 0.08 ^b^	0.70 ± 0.15 ^b^	0.64 ± 0.39 ^b^
Peak 11	29.92		0.06 ± 0.01 ^a^	0.81 ± 0.13 ^b^	0.58 ± 0.12 ^b^	0.50 ± 0.16 ^b^	0.67 ± 0.07 ^b^	0.66 ± 0.18 ^b^	0.66 ± 0.39 ^b^
Peak 12	34.13		2.64 ± 0.08 ^a^	5.24 ± 0.60 ^bc^	5.06 ± 0.21 ^bc^	5.30 ± 0.11 ^bc^	5.55 ± 0.63 ^c^	4.92 ± 0.01 ^bc^	4.44 ± 0.05 ^b^
Peak 13	41.91		5.19 ± 0.34 ^a^	7.13 ± 0.46 ^c^	6.25 ± 0.10 ^b^	6.77 ± 0.39 ^bc^	6.84 ± 0.23 ^bc^	6.37 ± 0.04 ^b^	6.53 ± 0.31 ^b^
Peak 14	45.29		13.03 ± 0.28 ^a^	16.73 ± 1.57 ^bc^	15.85 ± 0.15 ^b^	16.77 ± 0.03 ^bc^	17.38 ± 0.36 ^c^	16.19 ± 0.02 ^bc^	16.55 ± 0.36 ^bc^
		Total	29.00 ± 0.73 ^a^	49.62 ± 2.11 ^c^	49.76 ± 1.59 ^c^	46.68 ± 1.82 ^b^	49.55 ± 1.52 ^c^	47.58 ± 0.49 ^bc^	46.69 ± 2.56 ^c^

Data are expressed as g/100 g dry material. Mean values ± standard deviation (*n* = 4). Different superscript letters in each raw denote significant differences. RT: Retention time. Nd: Not detected. Regression equations used: Peaks 1–5 were estimated as polysaccharides or high molecular weight carbohydrates (HMWC). Peaks 6–14 were estimated as oligosaccharides and simple sugars or low molecular weight carbohydrates (LMWC).

**Table 2:** HPLC-RID analysis of water-soluble carbohydrates released from apple by-product after HHP treatment non-assisted by Celluclast^®^.

Sample	200 MPa		400 MPa		600 MPa	
Time (min)	15	30	15	30	15	30
Peak 1	7.88 ± 0.16 ^b^	8.93 ± 1.27 ^b^	8.69 ± 0.74 ^b^	8.71 ± 0.99 ^b^	8.02 ± 0.68 ^b^	9.52 ± 0.61 ^b^
Peak 2	3.29 ± 0.12 ^a^	3.53 ± 0.40 ^a^	3.24 ± 0.22 ^a^	3.51 ± 0.21 ^a^	3.10 ± 0.20 ^a^	3.24 ± 0.06 ^a^
Peak 3	0.70 ± 0.06 ^b^	0.82 ± 0.11 ^b^	0.78 ± 0.07 ^b^	0.86 ± 0.06 ^b^	0.70 ± 0.11 ^b^	0.76 ± 0.07 ^b^
Peak 8	0.33 ± 0.02 ^a^	0.33 ±0.02 ^a^	0.28 ± 0.01 ^a^	0.35 ± 0.05 ^a^	0.33 ± 0.03 ^a^	0.35 ± 0.03 ^a^
Peak 10	0.21 ± 0.01 ^a^	0.21 ± 0.01 ^a^	0.20 ± 0.02 ^a^	0.21 ± 0.02 ^a^	0.21 ± 0.01 ^a^	0.22 ± 0.02 ^a^
Peak 11	0.10 ± 0.01 ^a^	0.11 ± 0.02 ^a^	0.11 ± 0.02 ^a^	0.13 ± 0.06 ^a^	0.07 ± 0.01 ^a^	0.13 ± 0.05 ^a^
Peak 12	2.77 ±0.17 ^a^	2.91 ± 0.12 ^a^	2.60 ± 0.23 ^a^	2.69 ± 0.45 ^a^	2.37 ± 0.36 ^a^	2.29 ± 1.30 ^a^
Peak 13	6.09 ± 0.18 ^b^	6.12 ± 0.06 ^b^	6.51 ± 0.20 ^b^	6.39 ± 0.43 ^b^	6.53 ± 0.23 ^b^	6.32 ± 0.28 ^b^
Peak 14	15.07 ± 0.27 ^b^	15.27 ± 0.09 ^b^	15.58 ± 0.11 ^b^	15.39 ± 0.64 ^b^	15.53 ± 0.39 ^b^	15.21 ± 0.37 ^b^
Total	36.19 ± 1.05 ^b^	37.35 ± 2.27 ^b^	37.96 ± 0.52 ^b^	37.50 ± 0.74 ^b^	36.04 ± 1.42 ^b^	37.90 ± 0.80 ^b^

Data are expressed as g/100 g dry material. Mean values ± standard deviation (*n* = 4). Different superscript letters in each raw denote significant differences. Regression equations used: Peaks 1–3 were estimated as polysaccharides or high molecular weight carbohydrates (HMWC). Peaks 8–14 were estimated as oligosaccharides and simple sugars or low molecular weight carbohydrates (LMWC).

**Table 3:** Linearity, sensitivity, molecular weight estimation and precision of carbohydrates standards by HPLC-RID.

Standard	MW (kDa)	RT (min)	Linearity (*R*^2^)	Sensitivity		Estimated MW ^a^ (kDa)	LOD ^b^ (µg/mL)	LOQ ^c^ (µg/mL)	Repeatability RSD (%)	Inter-Day Precision RSD (%)
				Slope (m)	Intercept (b)					
Pullulan 100	100	14.99	0.999	1.032	1.957	104.06	1.81	6.02	0.01	0.01
Pullulan 50	50	15.18	0.999	1.033	2.166	40.09	1.23	4.09	0.01	<0.01
Pullulan 20	20	15.31	0.998	1.054	1.963	20.88	1.68	5.59	0.01	0.01
Pullulan 10	10	15.40	0.999	0.991	2.130	13.29	1.82	6.08	0.01	0.04
Inulin	5.94	15.59	0.999	1.068	1.519	5.12	1.22	4.07	0.01	0.06
Verbascose	0.83	23.40	0.999	0.948	2.207	0.77	3.25	10.83	<0.01	0.01
Stachyose	0.67	25.93	0.999	1.044	1.947	0.64	3.32	11.08	0.01	0.02
Cellotriose	0.50	28.10	0.999	1.041	1.943	0.55	3.33	11.11	0.01	0.03
Raffinose	0.50	29.58	0.999	1.017	1.966	0.49	3.29	10.96	0.01	0.02
Cellobiose	0.34	33.80	0.999	0.982	2.165	0.36	3.34	11.15	0.03	0.03
Sucrose	0.34	34.28	0.999	0.968	2.213	0.35	3.49	11.63	<0.01	<0.01
Glucose	0.18	41.93	0.999	0.911	2.461	0.20	3.51	11.70	<0.01	<0.01
Fructose	0.18	45.34	0.999	0.936	2.364	0.16	3.52	11.72	0.03	0.03

Data are mean values ± standard deviation (*n* = 3). RT: Retention time. RSD: Relative standard deviation. ^a^ Regression equation used: Log (MW) = −2.1799 RT + 37.694 for polysaccharides or high molecular weight carbohydrates (HMWC). Log (MW) = −0.0315 RT + 3.623 for oligosaccharides and simple sugars or low molecular weight carbohydrates (LMWC). ^b^ LOD: Signal-to-noise ratio equals 3. ^c^ LOQ: Signal-to-noise ratio equals 10.
